# High hepatitis C virus seropositivity, viremia, and associated risk factors among trans women living in San Francisco, California

**DOI:** 10.1371/journal.pone.0249219

**Published:** 2021-03-30

**Authors:** Christopher J. Hernandez, Dillon Trujillo, Sofia Sicro, Joaquin Meza, Mackie Bella, Emperatriz Daza, Francisco Torres, Willi McFarland, Caitlin M. Turner, Erin C. Wilson

**Affiliations:** 1 Center for Public Health Research, San Francisco, CA, United States of America; 2 Department of Epidemiology and Biostatistics, University of California, San Francisco, United States of America; Centers for Disease Control and Prevention, UNITED STATES

## Abstract

Trans women have been understudied in the Hepatitis C virus (HCV) epidemic, yet data suggest they may be at elevated risk of the disease. Using data collected from the Centers for Disease Control and Prevention’s (CDC) National HIV Behavioral Surveillance (NHBS) survey, we measured HCV seropositivity, viremia, and associated risk factors for HCV infection among trans women in San Francisco from June 2019 to February 2020. Respondent-driven sampling (RDS) was used to obtain a diverse, community-based sample of 201 trans women, of whom 48 (23.9%, 95% CI 17.9% - 30.0%) were HCV seropositive. HCV seropositivity significantly increased with increasing age (adjusted prevalence ratio [APR] 1.04 per year, 95% CI 1.01–1.07) and history of injection drug use (APR 4.44, 95% CI 2.15–9.18). We also found that many had HCV viremia as twelve (6.0% of the total sample, 95% CI 2.7% - 9.3%) were RNA-positive for HCV. Trans women are highly impacted by HCV and could benefit from access to regular and frequent HCV screening and treatment access. HCV screening could be offered regularly in trans-specific health services, in the community, in jails and prisons, and integrated syringe exchange programs where treatment access or referral are also available.

## Introduction

Hepatitis C Virus (HCV) is a pathogen of the liver that is most often transmitted through contact with infected blood [1]. Although nearly 20% of new infections are resolved spontaneously, the majority become chronic infections that can lead to cirrhosis and hepatocellular carcinoma, underscoring the importance in identifying and treating people with HCV infection [2]. Detecting HCV is not straightforward. Rapid tests, which typically have a 6 to 9-week window period, are used to determine a person’s HCV seropositivity, or the presence of anti-HCV antibodies made in response to infection [3,4]. An exposed person’s seropositive status typically remains even after infection has resolved spontaneously or has been successfully treated; therefore, people who test HCV seropositive may not have an active infection, necessitating a screen for HCV viremia, or the detection of HCV viral RNA in blood, to determine chronicity [4,5]. Lastly, some people with an HCV infection may test HCV seronegative because they have not yet mounted an antibody response, thus HCV RNA tests, which have a window period of 1 to 2 weeks, can detect early infections with HCV, but are not usually employed for this purpose [6,7]. Novel direct acting antivirals are highly successful at eliminating HCV infection suggesting that, combined with other prevention measures, HCV can be eliminated as a public health threat if people living with HCV viremia are found and engaged in treatment [8].

A steady decline in HCV incidence occurred between 1990 and 2005, a trend attributable to better screening of donated blood products and a decrease in injection drug use [9]. Prior to screening improvements, people born between 1946 and 1964, known as the baby boomer generation, were the most at risk for HCV and represented 74% of all chronic HCV cases [10]. However, recent studies show HCV risk increasing among young people, with people between the ages of 20–39 years old being overrepresented in newly reported chronic HCV infections compared to the general population [11]. Additionally, there was a tripling of HCV cases in the United States between 2011 and 2016, mostly impacting people who inject drugs (PWID), people who are incarcerated, those who experience homelessness, and communities facing significant social marginalization [12–14]. To date, trans women have been understudied in the HCV epidemic, but emerging data suggest they may also be at elevated risk [15].

Trans women experience several factors known to put them at risk for HIV that may also increase their risk for HCV infection [16,17]. Among these are high rates of homelessness, substance use, and incarceration, which are factors independently associated with HCV infection risk [18]. Approximately 65% of long-term injection drug users and 35% of short-term injection drug users were infected with HCV in the United States [19]. Nearly 46% of people experiencing homelessness in San Francisco have been infected with HCV [20]. Within prisons in the United States, 62.5% of women injected drugs, highlighting the role of prisons in concentrating and amplifying HCV risk [21,22]. A study from 2016 in San Francisco found that 23.8% of trans women were HCV seropositive [23]. We know of no studies that have documented prevalence of viremia among trans women or studies to determine through multivariable models what factors are independently associated with increased HCV risk.

This study was conducted to fill gaps in what we know about HCV among trans women. Using community-based data collected with trans women, we obtained an estimate for HCV seropositivity and determined the correlates of HCV risk. We also examined data to determine how many trans women had an active HCV infection.

## Materials and methods

This was a secondary analysis of data collected from the San Francisco site of the National HIV Behavioral Surveillance Transgender Women (NHBST) study. It was the first survey among trans women conducted as part of the National HIV Behavioral Surveillance Study (NHBS) cycles led by the Centers for Disease Control and Prevention (CDC). The survey questionnaire used in this study can be found at the CDC NHBS website for additional populations [24]. The primary objectives of the NHBS were to measure HIV prevalence and use of HIV prevention and care services [25,26]. The local implementation of the NHBST study offered HIV, STI and HCV serological and nucleic acid testing. Demographic and behavioral data originate from the NHBS core questionnaire and supplemental questions implemented in San Francisco.

### Participants, sampling, and recruitment

Recruitment and sampling methods were identical to three previous local surveys of trans women conducted in 2010, 2013, and 2016 [27,28] with the addition of a screen for HCV viremia in the current wave. Briefly, respondent-driven sampling (RDS) was used to obtain a diverse, community-based sample of trans women. Twenty-five trans women who previously participated in other studies done in San Francisco, from socially diverse networks with respect to demographic characteristics such as age, race/ethnicity, and educational level, were enlisted as “seeds” to recruit their peers. Discussions were held with principal investigators and the community advisory board to determine if seeds were appropriately selected for target networks. These initial 25 “seeds” were instructed to refer 3 to 5 other trans women from their networks who were 18 years of age or older, resident of San Francisco, self-identified as a trans woman (i.e., as a woman or gender other than male as assigned at birth) and were fluent in English or Spanish. Eligible recruits provided written and verbal consent, completed an interviewer-administered survey, and were offered rapid HIV HCV, and STI testing. Blood specimens were provided for the detection of HIV and HCV antibodies. Detection of HCV antibodies is hereafter referred to as HCV seropositivity. Blood samples were also used for the screening of HCV viral RNA, with positive samples from this point forward referred to as HCV viremia. Participants received a total of $100 for completing the study activities and an additional $25 for each eligible peer referral enrolled into the study. Recruitment continued until the sample size (N = 201) was met, and the composition of the sample stabilized with respect to demographic characteristics.

### Measures

Participants were asked about their demographic details, such as age, race/ethnicity, education, income, and housing. They were also asked about behavioral patterns, such as sexual behavior, substance use, and access to healthcare. Participants were considered HCV seropositive if they tested positive using a rapid Oraquick® HCV Rapid Antibody Test (OraSure Technologies, Bethlehem, PA) that ran for 20 minutes. Participants were considered viremic if HCV RNA was detected during lab-based blood testing conducted at the local San Francisco Department of Public Health laboratory. Participants were considered HIV positive if they tested positive using a rapid HIV test using Oraquick® HIV Rapid Antibody Test (OraSure Technologies, Bethlehem, PA). HCV and HIV tests were offered to participants after completion of the interview.

### Statistical analysis

We conducted analyses using STATA version 15 (College Station, TX). Descriptive statistics (i.e., proportions) were used to summarize demographic characteristics, behaviors, and HCV seropositivity by these variables. The chi-square or Fisher’s exact tests were used to test differences in HCV seropositivity by demographic and risk subgroups (i.e., age, race, education, income, PWID, history of incarceration etc.) using ≤ 0.05 as the level for significance. To find independent correlates between demographic and risk factors with HCV seropositivity, we built a multivariable model calculating prevalence ratios given the high seropositivity of HCV in the sample. In addition to demographic variables, such as age, race, and education, we included a history of injection drug use as it is a well-studied transmission route. We also included a history of incarceration as it has been observed to be correlated with HCV transmission [29], and HIV as it shares a syndemic interaction with HCV infection [30].

### Ethical considerations

Participants who tested positive for HIV, HCV, or other STIs were provided a brief counseling session and resources for linkage to care and treatment. The protocol was reviewed and approved by the Institutional Review Board (IRB) at the University of California, San Francisco (UCSF) (#17–24062). All participants provided informed consent.

## Results

### Participant characteristics

The study recruited 201 trans women who were diverse with respect to race/ethnicity and age (Table 1). Most were trans women of color, with 37.3% Hispanic/Latinx, 20.9% Black/African American, 8.9% Asian or Pacific Islander, 14.9% multiple or mixed race, and 17.9% non-Hispanic White. The mean age was 44.5 years (SD 11.8). A majority had an education of high school/GED or less (53.2%). One third (34.4%) reported college level education and 12.4% reported having a bachelor’s degree or higher. A majority (57.7%) also had an income below the federal poverty line and 59.7% experienced homelessness in the past 12 months. Most (92.5%) had medical insurance at the time of the survey. Many respondents (89.1%) accessed trans-specific health and social services in San Francisco in 2018.

**Table 1:** Characteristics of trans women in the National HIV Behavioral Surveillance (NHBS) survey by hepatitis C virus (HCV) antibody status, San Francisco, CA, 2019 (N = 201).

Characteristic	Total (N = 201)	HCV-antibody positive n (%)^1^	HCV-antibody negative n (%)	p-value^2^
Race/Ethnicity				
Non-Hispanic White	36 (17.9)	13 (36.1)	23 (63.8)	0.06
Black/African American	42 (20.9)	15 (35.7)	27 (64.3)	
Hispanic/Latinx	75 (37.3)	13 (17.3)	62 (86.1)	
Asian Pacific Islander	18 (9.0)	2 (11.1)	16 (88.9)	
Multiple races/other	30 (14.9)	5 (16.7)	25 (83.3)	
Age group in years				
18–29	28 (13.9)	0 (0.0)	28 (100)	
30–39	42 (20.9)	4 (9.5)	38 (90.5)	<0.001
40–49	52 (25.9)	10 (19.2)	42 (80.8)	
50 and above	79 (39.3)	34 (43.0)	45 (57.0)	
Education				
High school diploma or less	107 (53.2)	31 (29.0)	76 (71.0)	0.189
College level education	69 (34.3)	12 (17.4)	57 (82.6)	
Bachelor’s degree or higher	25 (12.4)	5 (20.0)	20 (80.0)	
Income				
Below federal poverty	116 (57.7)	29 (25.0)	87 (75.0)	0.664
Above federal poverty	85 (42.3)	19 (22.3)	66 (77.7)	
Currently homeless				
No	147 (73.1)	33 (22.4)	114 (77.6)	0.432
Yes	54 (26.9)	15 (27.8)	39 (72.2)	
Homeless in the past 12 months				
No	81 (40.3)	19 (23.5)	62 (76.5)	0.908
Yes	120 (59.7)	29 (24.2)	91 (75.8)	
Currently insured				
No	15 (7.5)	3 (20.0)	12 (80.0)	0.714
Yes	186 (92.5)	45 (24.2)	141 (75.8)	
Used trans specific health and social services in 2018				0.772
No	22 (10.9)	4 (18.2)	18 (81.8)	
Yes	179 (89.1)	43 (24.0)	136 (76.0)	
HIV PrEP use in the last 12 months^3^				
No	64 (55.2)	14 (21.9)	50 (78.1)	0.015
Yes	52 (44.8)	3 (5.8)	49 (92.5)	
Ever injection drug use				
No	139 (69.2)	11 (7.9)	128 (92.1)	
Yes	62 (30.8)	37 (59.7)	25 (40.3)	<0.001
Non-injection drug use (NIDU) in the past 12 months				
No	65 (32.3)	16 (24.6)	49 (75.4)	0.866
Yes	136 (67.7)	32 (23.5)	104 (76.5)	
Methamphetamine past 12 months				
No	137 (68.2)	28 (20.4)	109 (79.6)	0.094
Yes	64 (31.8)	20 (31.3)	44 (68.7)	
Crack cocaine past 12 months				
No	176 (87.6)	37 (21.0)	139 (79.0)	0.012
Yes	25 (12.4)	11 (44.0)	14 (56.0)	
HIV status				
Negative	116 (57.7)	17 (14.7)	99 (85.3)	<0.001
Positive	85 (42.3)	31 (36.5)	54 (63.5)	
Ever arrested or detained				
No	66 (32.8)	5 (7.6)	61 (92.4)	<0.001
Yes	135 (67.2)	43 (31.9)	92 (68.1)	

^1^Percentage is number of antibody positive over count of left row.

^2^chi-square or Fisher’s exact test.

^3^Restricted to participants not living with HIV.

### Hepatitis C seropositivity

Overall, 48 (23.9%, 95% CI 17.9% - 30.0%) trans women were HCV seropositive. HCV seropositivity was highest among older trans women, with 43.0% of trans women aged 50 or older testing seropositive for HCV, while HCV seropositivity was 19.2% for those aged 40–49 years, 9.5% for those aged 30–39 years, and no HCV seropositive cases among those aged 18–29 years (Table 1). More than one third (35.7%) of trans women who identified as Black/African American, 17.3% who identified as Hispanic/Latinx, 36.1% who identified as non-Hispanic white, 16.7% who identified as mixed or multiple races, and 11.1% who identified as Asian Pacific Islander tested HCV seropositive. By education, 29.0% of trans women with a high school diploma or less tested HCV seropositive, while 20.0% who reported having a bachelor’s degree or more, and 17.4% of those with some college tested HCV seropositive. Additionally, 25.0% of those who reported an income below the federal poverty line and 24.2% who reported homelessness in the last twelve months tested HCV seropositive. Among those who reported being arrested or detained by law enforcement at some point in their lives, 31.9% tested HCV seropositive.

Trans women who reported PrEP use in the last 12 months (5.8%) were significantly less likely to be HCV seropositive than those who did not report PrEP use (21.9%) (p = 0.015). HCV seropositivity was significantly associated with living with HIV, with 36.5% of those living with HIV were also HCV seropositive compared to 14.7% HCV seropositivity among those not living with HIV (p<0.001). Significantly more trans women who reported a lifetime history of injection drug use were HCV seropositive (59.7%) compared to those who did not report injection drug use (7.9%) (p < 0.001).

### Correlates of hepatitis C seropositivity

After controlling for demographic factors and other known risk factors, HCV seropositivity was significantly higher with increasing age (adjusted prevalence ratio [APR] 1.04 per year, 95% CI 1.01–1.07) (Table 2). Those with a history of injection drug use (APR 4.44, 95% CI 2.15–9.18) had a significantly higher HCV seropositivity than those who had no history of injection drug use.

**Table 2:** Independent associations with hepatitis C seropositivity among trans women in the National HIV Behavioral Surveillance (NHBS) survey, San Francisco, CA, 2019 (N = 201).

Characteristic	Adjusted Prevalence Ratio (95% CI)	p-value
Age in years	1.04 (1.01–1.07) per year	0.011
Race/Ethnicity		
Hispanic/Latinx	Reference	-
Non-Hispanic White	1.41 (0.62–3.21)	0.412
Black/African American	1.00 (0.46–2.19)	0.991
Asian Pacific Islander	0.75 (0.16–3.51)	0.713
Other/Mixed	0.74 (0.26–2.08)	0.563
Education		
High School or Less	Reference	-
Some College	0.77 (0.38–1.56)	0.467
Bachelors or higher	0.84 (0.30–2.33)	0.734
Living with HIV		
No	Reference	-
Yes	1.51 (0.79–2.89)	0.209
Ever arrested or detained		
No	Reference	-
Yes	1.85 (0.67–5.13)	0.235
Ever injection drug use		
No	Reference	-
Yes	4.44 (2.15–9.18)	0.001

#### Characteristics and HCV healthcare among those with HCV viremia

We screened 179 participants for the presence of HCV viremia. The remaining participants did not consent to the test or the phlebotomist was unable to perform a venipuncture. A total of 12 participants, or 6.0% of the total study participants were living with HCV viremia (Table 3). Eleven (92%) of those 12 individuals used injection drugs in their lifetime and nine (75%) injected in the last twelve months. Eleven (92%) reported being arrested or detained by police at some point in their lifetime. Seven (58%) were currently experiencing homelessness and 11 (92%) experienced homelessness in the past 12 months Seven (58%) of those who were living with HCV viremia were living with HIV. Four (33%) required healthcare in the past 12 months but were unable to afford it.

**Table 3:** Characteristics among trans women living with HCV viremia.

Characteristic	n = 12	%
Age group in years		
40–49	4	33
50 and above	8	67
Homeless past 12 months	11	92
Currently homeless	7	58
Incarceration	11	92
Life-time injection drug use	11	92
IDU last 12 months	9	75
Non-injection drug use	9	75
Living with HIV	7	58
Healthcare visit in the past 12 months	12	100
Needed healthcare in past 12 months but could not afford it	4	33

Among the 12 participants with HCV viremia, 11 (92%) were HCV seropositive (Table 4). The participant who was not seropositive had been screened for HCV in the past but not diagnosed with HCV previously, thus we may have observed an acute HCV infection. Three quarters (75%) had been diagnosed with HCV in the past but only 1 (12%) reported receiving HCV treatment, which was not completed. Two (17%) reported having cleared their first infection but acquired another HCV infection. No details about screening location or treatment site were disclosed.

**Table 4:** HCV care indicators among trans women with HCV viremia.

HCV care Indicator	n = 12	%
HCV-antibody present^1^	11	92
Prior HCV screening	11	92
Prior diagnosis of HCV infection	9	75
Aware of active HCV infection	9	75
Received HCV treatment	1	12
Completion of HCV treatment	0	0
Reinfection with HCV	2	17

^1^ Determined with Oraquick® HCV Rapid Antibody Test.

## Discussion

We found that trans women bear a high burden of HCV infection and should be prioritized in efforts to eliminate HCV. Nearly one fourth of trans women were HCV seropositive, a figure nine times higher than the prevalence of HCV in the general population of San Francisco [31]. We also found a high prevalence of HCV viremia with a relatively high awareness of HCV infection but little to no engagement in HCV treatment.

The strongest independent association with HCV seropositivity among trans women in our survey was a history of injection drug use. Trans women with a history of injection drug use had over four times the odds of infection compared to those with no injection history. Additionally, half of the trans women who had HCV viremia reported recent injection drug use, and a majority reported injecting drugs in their lifetime. Injection drug use was common with nearly one in three trans women having used injections drugs, which is consistent with prior research findings that injection drug use is a common coping strategy among trans women who face multiple sources of threats to their survival [32]. These findings indicate the importance of harm reduction services like syringe exchange for trans women to prevent acquisition of HCV and other infectious diseases like HIV. Syringe exchange availability in trans-specific clinics and social services that are standard of care could significantly reduce the HCV burden among trans women.

Older age was also found to be independently associated with HCV seropositivity in our study. The HCV epidemic among older trans women reflects the U.S. HCV epidemic overall in which the estimated 2.4 million cases of HCV in the United States occur mostly among those born between 1945–1965 [33]. Further, in our study no participants under the age of 30 were seropositive for HCV. Younger trans women may be less likely to have a history of injection drug use, or those who inject drugs may have more information and access to HCV screening, clean needles via syringe exchange programs and access to new highly tolerable HCV treatments, perhaps explaining lower rates of HCV among younger trans women.

Rapid HCV screens are an acceptable and important intervention strategy for identifying HCV infections among PWID [34] and increasing rapid HCV screening availability among trans women may help tackle the HCV epidemic, however our data suggests that focusing on screening efforts alone may lead to shortfalls. Three-quarters of our participants with HCV viremia in our study knew of their status but only one reported receiving treatment, which was not successfully completed. Efforts to recruit and retain trans women with HCV viremia in treatment should be emphasized. A randomized control trial among PWID in San Francisco found that recruiting active PWID to treatment sites was feasible and efficacious in treating HCV [35]. Interventions to increase HCV screening and treatment awareness and access could be situation in trans-specific healthcare sites or needle-exchange sites frequented by trans women.

Our study is not without limitations. First, as a secondary analysis of a survey on HIV, there were few specific questions on barriers to explain the low HCV treatment initiation and engagement. Second, our sample does not represent all trans women in San Francisco. As presented elsewhere [36] sampling of trans women for studies may be more likely to reach the lower income part of the population for a various reasons, including a bias toward low income people who may not be employed during the traditional workday making it easier to participate during those hours and the attractiveness of an incentive among people who have less money. Nonetheless, the RDS recruitment achieved a diverse sample of this vulnerable population and included persons not necessarily accessing clinics or engaged by outreach efforts. Despite the limitations, this study provides data on HCV seropositivity among a community of trans women living in San Francisco. HCV seropositivity correlates and indicators of screening and treatment give direction to research needed on this health issue in order to eliminate infection from this disproportionately affected population. As HCV is controlled in other groups with fewer barriers to care, the relative burden may become even more pronounced among trans women. RDS or other peer-referral methodology to sample trans women may also provide a means to provide screening and treatment to those who are not reached by conventional programs and outreach efforts.

## Conclusions

Our study showed that trans women are highly impacted by HCV as a population, with some awareness of their infections but notably low HCV treatment engagement. Some trans women knew they were living with HCV, while others did not know, and others had previously been infected with HCV and were now re-infected and did not know their status. All the trans women who were viremic have had a healthcare visit in the last year. There is a need for more regular HCV screening among trans women during their healthcare visits. Many trans women in our study also experienced incarceration and HCV was associated with injection drug use. HCV screening and treatment access in jails, prisons, syringe exchange sites and other places where people who inject drugs seek services may reach trans women at risk of HCV. Studies are also needed to determine what are the barriers to HCV screening and why trans women are not engaged in HCV treatment. While HIV is often a focus of research with trans women, other infectious disease research may need to be expanded to meet their unmet health needs.
